# Supplementary material for: Structure–Activity Relationships of Low Molecular Weight Alginate Oligosaccharide Therapy against Pseudomonas aeruginosa
Source: Biomolecules. 2023 Sep 8;13(9):1366. doi: 10.3390/biom13091366 (PMC10527064; doi:10.3390/biom13091366)
Supplement: Supplementary file 1 [file biomolecules-13-01366-s001.zip › biomolecules-2505419-supplementary.pdf]

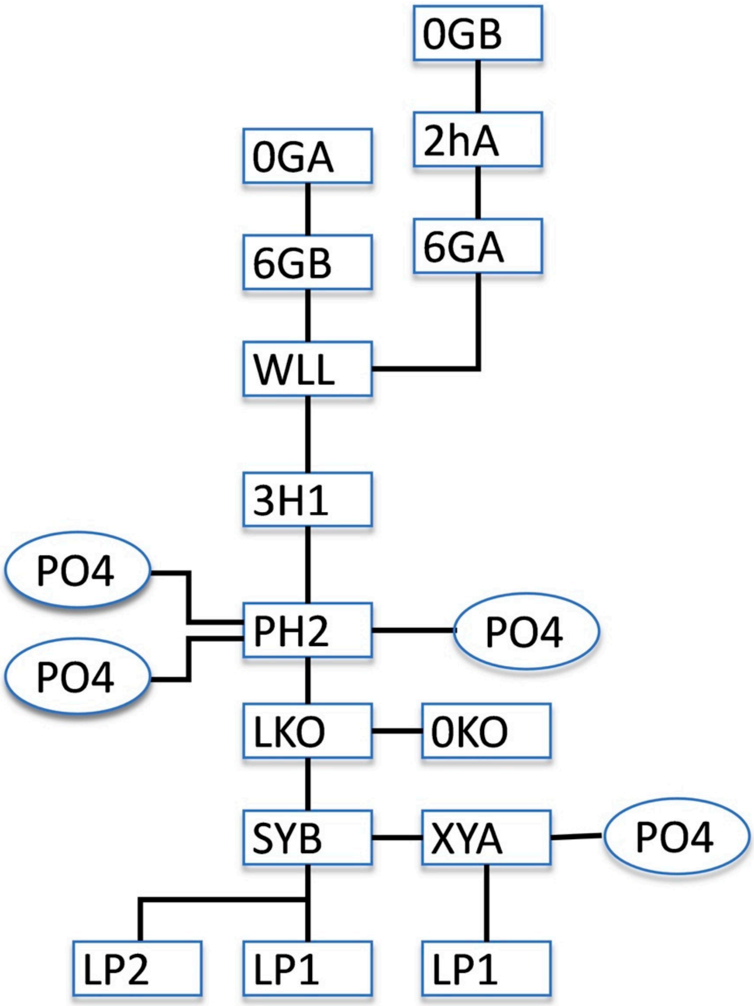

Supplementary Figure S1: schematic representation of potential LPS binding sites during MD simulations

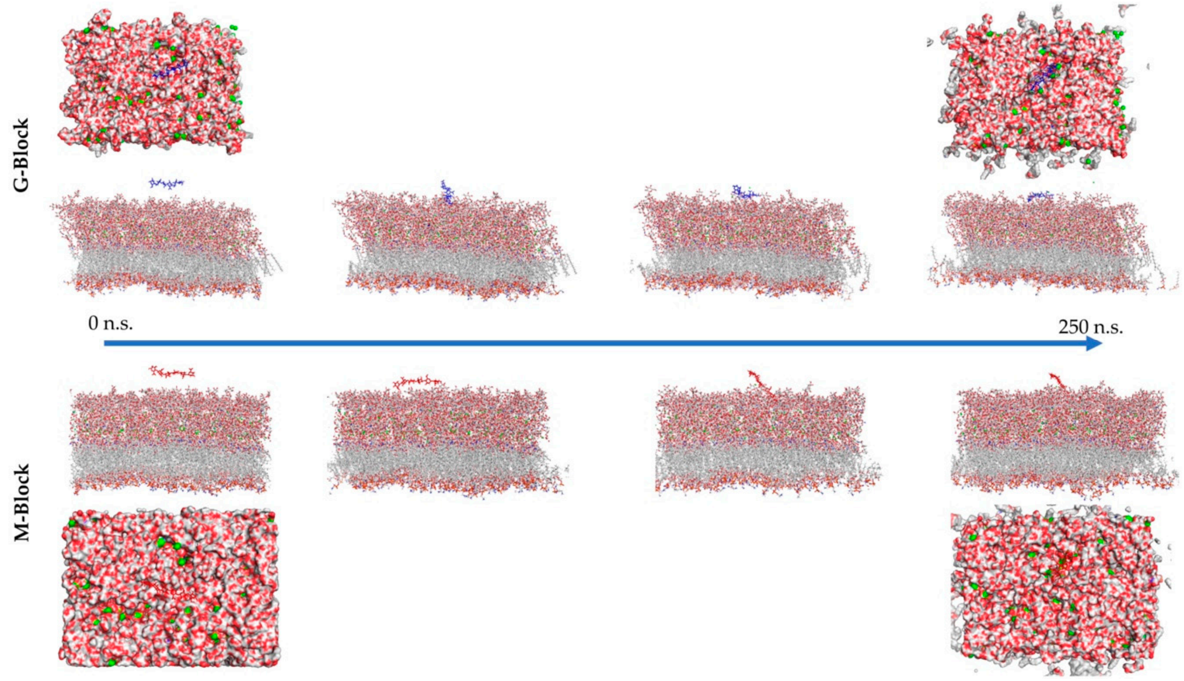

Supplementary Figure S2: representative (side-view) images of the G-block and M-block simulations taken at equal intervals between 0 and 250 ns (Ca^{2+} ions are shown in orange), with arial view images at the start and end of the simulation also shown.
